# Purification and Characterization of a Novel Cold Shock Protein-Like Bacteriocin Synthesized by *Bacillus thuringiensis*

**DOI:** 10.1038/srep35560

**Published:** 2016-10-20

**Authors:** Tianpei Huang, Xiaojuan Zhang, Jieru Pan, Xiaoyu Su, Xin Jin, Xiong Guan

**Affiliations:** 1 State Key Laboratory of Ecological Pest Control for Fujian and Taiwan Crops & Key Laboratory of Biopesticide and Chemical Biology (Ministry of Education), College of Life Sciences, Fujian Agriculture and Forestry University, Fuzhou 350002, Fujian, China; 2 Fujian-Taiwan Joint Center for Ecological Control of Crop Pests, Fuzhou 350002, Fujian, China; 3 Fuzhou Center for Disease Control and Prevention, Fuzhou 350004, Fujian, China

## Abstract

*Bacillus thuringiensis* (Bt), one of the most successful biopesticides, may expand its potential by producing bacteriocins (thuricins). The aim of this study was to investigate the antimicrobial potential of a novel Bt bacteriocin, thuricin *Bt*CspB, produced by Bt BRC-ZYR2. The results showed that this bacteriocin has a high similarity with cold-shock protein B (CspB). *Bt*CspB lost its activity after proteinase K treatment; however it was active at 60 °C for 30 min and was stable in the pH range 5–7. The partial loss of activity after the treatments of lipase II and catalase were likely due to the change in *Bt*CspB structure and the partial degradation of *Bt*CspB, respectively. The loss of activity at high temperatures and the activity variation at different pHs were not due to degradation or large conformational change. *Bt*CspB did not inhibit four probiotics. It was only active against *B. cereus* strains 0938 and ATCC 10987 with MIC values of 3.125 μg/mL and 0.781 μg/mL, and MBC values of 12.5 μg/mL and 6.25 μg/mL, respectively. Taken together, these results provide new insights into a novel cold shock protein-like bacteriocin, *Bt*CspB, which displayed promise for its use in food preservation and treatment of *B. cereus*-associated diseases.

The increasing trend of limiting the use of chemical food preservatives has stimulated research in the field of biopreservation to find an attractive and alternative approach to chemical preservatives. Among the biopreservatives, bacteriocins are receiving attention due to their Generally Recognized as Safe (GRAS) status^1^. Bacteriocins, including antimicrobial peptides and proteins, are different from conventional antibiotics, which are generally considered secondary metabolites^2^,^3^. Bacteriocins are classified into four different groups^4^. Bacteriocins that are synthesized by lactic-acid-producing bacteria have been well studied. However, only commercially produced bacteriocins are Nisin (*Lactococcus lactis*) and Pediocin (*Pediococcus acidilactici*). Others are still in a process of getting commercial status to be used as food preservatives^5^,^6^.

*Bacillus thuringiensis* (Bt) is one of the most successful entomopathogenic bacteria that has been used as a biopesticide due to its ability to synthesize insecticidal crystal proteins (ICPs) during sporulation and vegetative insecticidal proteins (VIPs) during the vegetative phase of growth^5^,^7^,^8^,^9^. Bt can also produce a variety of bacteriocins, called thuricins, with known sequences and bacteriocin-like inhibitory substances (BLIS), but these substances have not yet been fully characterized^10^,^11^,^12^,^13^. Thus, Bt bacteriocins may expand the potential of this insecticide to other purposes^5^. A considerable number of Bt BLIS have been reported in the literature, but only three Bt bacteriocins (thuricin-17, thurincin H and thuricin CD) with known amino acids have been thoroughly characterized^5^,^14^. Hence, many bacteriocins remain to be explored for their potential in food preservation and applications in agriculture as well as human and animal health.

*Bacillus cereus* is a bacterium that causes food poisoning with emetic and diarrhoeal symptoms, and it is frequently found in a wide range of raw food materials, such as dairy products, bakery products, rice and seafood^15^. Because *B. cereus* is highly resistant to various stresses (heat, cold, radiation, desiccation, and disinfectants) and show excellent adhesion to food surfaces, *B. cereus* contamination is very difficult to control^16^. In this study, a novel bacteriocin that is active against *B. cereus* and produced by Bt BRC-ZYR2 isolated from uranium-contaminated soil^7^ was purified and characterized, and its antibacterial efficacy was evaluated.

## Results

### Screening of bacteriocin producers active against *B. cereus*

The screening of bacteriocin producers from 79 Bt strains (see Supplementary Table 1) led to the identification of six Bt strains active against *B. cereus* 0938 and 19 Bt strains toxic to *B. cereus* ATCC 10987. Of these, Bt BRC-ZYR2 exhibited the highest antibacterial activity of the five strains active against both *B. cereus* strains (see Supplementary Table 1).

### Kinetics of bacteriocin production

**Figure 1:**
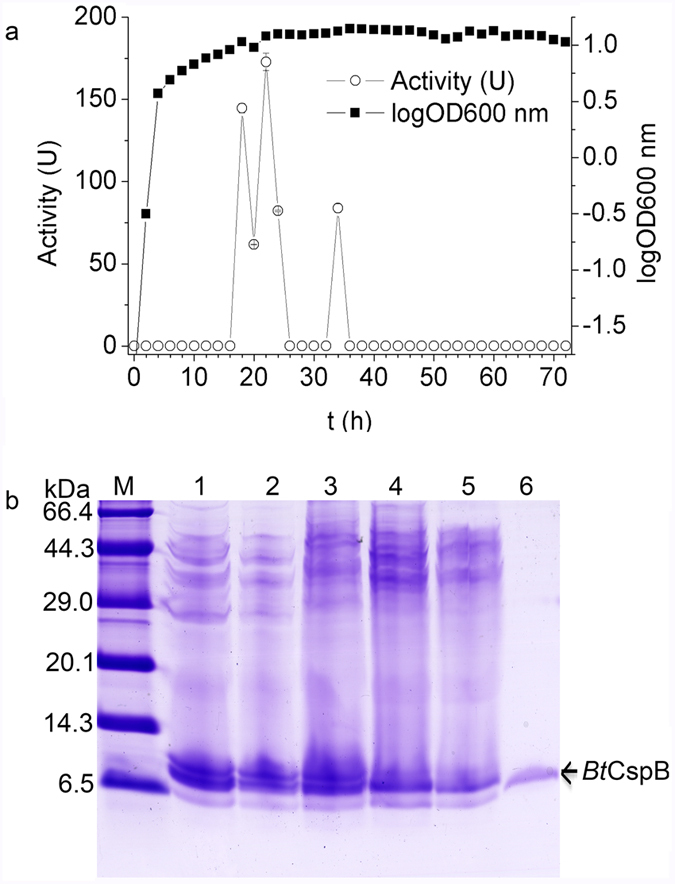
Kinetics of bacteriocin production by Bt BRC-ZYR2. (**a**) The correlation between the growth of Bt BRC-ZYR2 and the activities of its bacteriocins in TSB medium. The standard deviations were smaller than the size symbols used in most points. (**b**) The tricine-SDS-PAGE of the Bt BRC-ZYR2 supernatant samples with bacteriocin activity. Lane M: TaKaRa premixed protein marker (Broad). Lanes 1–5: Bt BRC-ZYR2 supernatant samples from 18 h, 20 h, 22 h, 24 h and 34 h, respectively. Lane 6: *Bt*CspB purified with the second gel filtration purification step using cellulose DEAE-52 anion-exchange chromatography.

Using *B. cereus* 0938 isolated from milk as the indicator strain, the bacteriocin production of Bt BRC-ZYR2 was found to start at the late logarithmic phase (18 h after incubation) in the growth cycle and continued until the beginning of the stationary phase (24 h after incubation). It was not active against *B. cereus* at 12 h after incubation like that of the above screening experiment. The bacteriocin activity re-appeared at 34 h after incubation. The highest level of bacteriocin activity was recorded during the late logarithmic phase (22 h after incubation) of growth in TSB at 30 °C (Fig. 1a). To demonstrate that the increase in antibacterial activity was due to an increase in the amount of bacteriocin produced by the strain and that the strain produced the same peptide at 34 h, the BLIS samples were further analyzed using a tricine sodium dodecylsulfate (SDS) polyacrylamide gel (tricine-SDS-PAGE). The results showed that the BLIS obtained 22 h and 34 h after incubation contained the same thuricin and that the concentration of the thuricin was higher at 22 h (Fig. 1b).

### Purification of Bt BRC-ZYR2 bacteriocins

**Figure 2:**
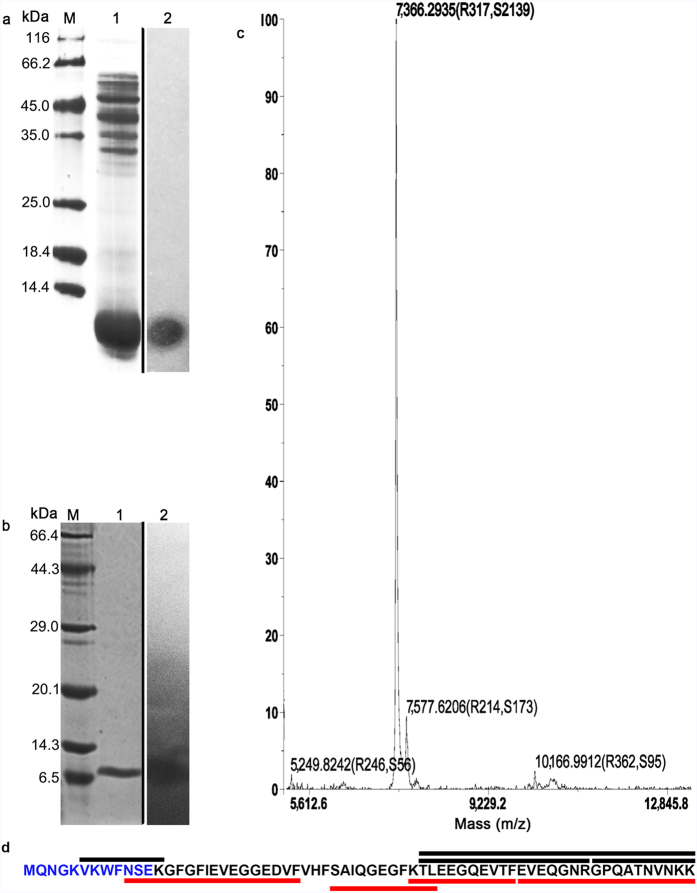
Determination of the primary structure of *Bt*CspB. (**a**) The tricine-SDS-PAGE of Bt BRC-ZYR2 bacteriocins and the detection of their antibacterial activity *in situ* on a gel. Lane M: Fermentas unstained protein molecular weight marker (SM0431). Lane 1: Bt BRC-ZYR2 bacteriocins precipitated by 100% ammonium sulfate. Lane 2: Antibacterial activity of Bt BRC-ZYR2 bacteriocins detected *in situ* on gel. The samples had been run in a same tricine-SDS-PAGE gel under the same experimental conditions. After electrophoresis, the gel was cut vertically. The first part containing a protein marker (Lane M) and the purified bacteriocins (Lane 1) were stained with Coomassie brilliant blue (CBB) R-250 to estimate the MW. The second part containing the purified bacteriocins (Lane 2) was assayed for the direct detection of bacteriocin activity by the presence of an inhibition zone. The borders of two parts were clearly demarcated in the figure by setting up a black line. (**b**) The protein profile of *Bt*CspB purified with a second round of gel filtration using cellulose DEAE-52 anion-exchange chromatography. Lane M: TaKaRa premixed protein marker (Broad). Lane 1: *Bt*CspB purified with a second round of gel filtration using cellulose DEAE-52 anion-exchange chromatography. Lane 2: Antibacterial activity of *Bt*CspB detected *in situ* on a gel. The samples had been run as (a). (**c**) The molecular mass determination of *Bt*CspB by AB SCIEX 5800 MALDI-TOF/TOF MS. A major peak at 7366.2935 Da represents the molecular ion peak. (**d**) The coverage map of *Bt*CspB by N-terminal amino acid sequencing and LC-MS/MS. The blue characters were the 12 N-terminal amino acids sequenced by Edman degradation (see Supplementary Fig. 4). The characters with a black overline corresponded to the tryptic peptides identified by LC-MS/MS (see Supplementary Fig. 5a), whereas the characters with a red underline represented the chymotryptic peptides identified by LC-MS/MS (see Supplementary Fig. 5b).

The pure bacteriocins of Bt BRC-ZYR2 were extracted by a four-step chromatographic process that used *B. cereus* 0938 as the indicator strain. In the initial step, the BLIS sample from Bt BRC-ZYR2 cultured at 30 °C for 22 h in 2 L TSB media was concentrated to 68 mL by 100% ammonium sulfate precipitation. After tricine-SDS-PAGE, CBB staining of the gel showed major bands at 30–55 KDa and <14.4 kDa (Fig. 2a). The gel used for the direct detection of antibacterial activity had a single and clear inhibition zone corresponding to a <14.4 kDa protein (Fig. 2a). The BLIS sample was loaded onto a Sephadex G-50 gel filtration column (φ10 × 600 mm). There were two chromatographic peaks, and the fractions between 78 min and 108 min (BLIS) in peak II (see Supplementary Fig. 1) were positive for antibacterial activity. These fractions were pooled and dialyzed overnight. Then, the BLIS sample was loaded onto a DEAE-52 Sepharose column (φ16 × 300 mm), and four peaks appeared. The active eluents of 167–188 min in peak IV (see Supplementary Fig. 2) were pooled and dialyzed overnight. They were loaded onto a clean DEAE-52 Sepharose column (φ16 × 300 mm) again. Of the five resulting peaks, the eluents of 64–94 min in peak II were active against *B. cereus* 0938 (see Supplementary Fig. 3). These fractions were pooled and dialyzed overnight. This active and pure bacterioncin of Bt BRC-ZYR2 appeared as one 7 kDa band on a tricine-SDS-PAGE gel (Fig. 2b), and was 35.6 μg/mL in a 2.5 mL solution (see Supplementary Table 3).

### Primary structure of *Bt*CspB

The molecular mass of the thuricin was found to be 7366.2935 Da by MALDI-TOF/TOF MS (Fig. 2c). Its 12 N-terminal amino acids were determined to be MQNGKVKWFNSE (Fig. 2d, also see Supplementary Fig. 4). Based on these results, the putative open reading frame (ORF) encoding the thuricin was derived from the draft genome sequence of Bt BRC-ZYR2 (unpublished data). The peptide consists of 66 amino acid residues (Fig. 2d) and has a predicted molecular weight of 7366.1, which is in agreement with the experimentally derived value of 7366.2935 Da by MALDI-TOF/TOF MS. The calculated isoelectric point for the peptide (4.84) was due to its high content (15.2%) of glutamic acid residues. With tryptic or chymotryptic digestion, *Bt*CspB was further confidently identified by liquid chromatography and tandem mass spectrometry (LC-MS/MS). Both digestions of the thuricin produced four detectable unique peptides and showed 53.03% and 81.82% coverage of the cold shock protein B (CspB) of Bt subsp. *konkukian* strain 97–27, respectively (Fig. 2d, also see Supplementary Fig. 5). Hence, the bacteriocin was designated as thuricin *Bt*CspB and deposited in the GenBank with an accession number ALM26172.

To investigate the coding gene distribution in Bt and *B. cereus* strains and their variation, *Bt*CspB gene was cloned (see Supplementary Fig. 6) and sequenced (GenBank accession number KT002356) based on the draft genome sequence of Bt BRC-ZYR2 (unpublished data). BLAST analysis revealed that Bt and *B. cereus* strains with completed genomes sequenced have one *Bt*CspB-like gene located at the chromosomes, which is 100% identity with *Bt*CspB gene.

**Figure 3:**
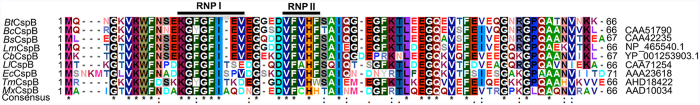
Multiple sequence alignment of the amino acid sequence of *Bt*CspB with other CspBs. The alignment was made with the ClustalW2 program. The identical residues present in the sequences are indicated with an asterisk, and the similar amino acids are indicated with points. The dashes represent gaps introduced to optimize the alignment. The highly conserved nucleic acid binding motifs (RNP I and RNP II) of the cold shock domain proteins are overlined. The abbreviations of the species are on the left, and their full names are as follows: *Bc (Bacillus caldolyticus*), *Bs (Bacillus subtilis*), *Lm (Listeria monocytogenes* EGD-e), *Cb (Clostridium botulinum* A strain ATCC 3502), *Ll (Lactococcus lactis* subsp. *cremoris* AM2), *Ec (Escherichia coli* strain TAP90), *Tm (Thermotoga maritima* MSB8), and *Mx (Myxococcus xanthus*). The GenBank accession numbers of the CspBs are on the right.

The alignment of the *Bt*CspB sequence with those of CspBs with a known cold shock function (Fig. 3) showed that *Bt*CspB shares 83.33%, 81.82% and 59.09% sequence identity with the CspBs from *Bacillus caldolyticus (Bc*CspB)*, Bacillus subtilis (Bs*CspB) and *Escherichia coli (Ec*CspB), respectively. Figure 3 also shows that two putative ribonucleoprotein (RNP) motifs, motifs I (K13-V20) and II (V26-F30) of the CSPs, were conserved in *Bt*CspB.

### Effects of enzymes, temperature and pH on the activity and stability of *Bt*CspB

**Figure 4:**
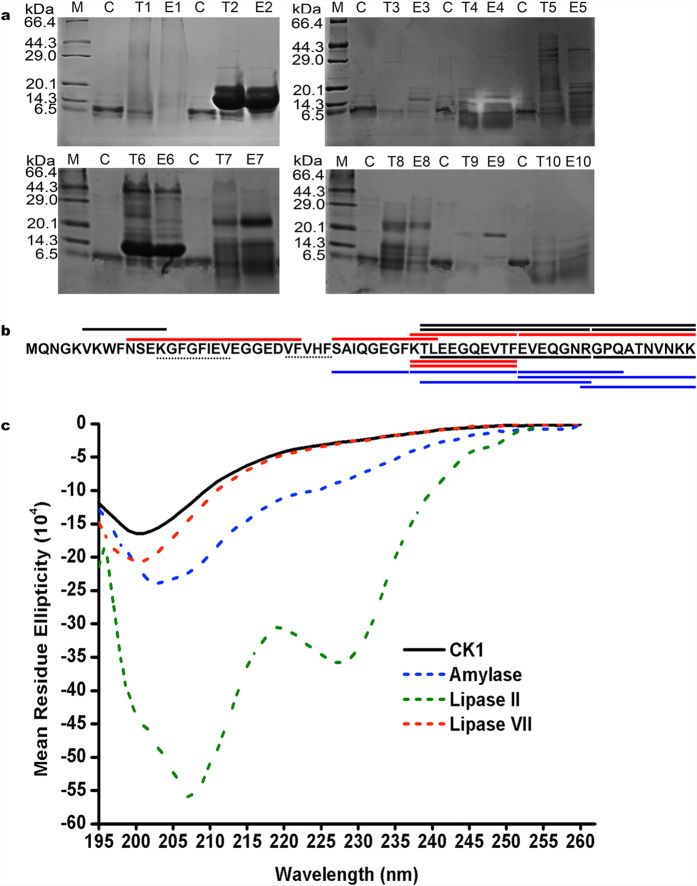
Stability of *Bt*CspB after treatment by different enzymes. (**a**) Tricine-SDS-PAGE. Lane M: TaKaRa premixed protein marker (Broad). Lane C: *Bt*CspB. Lanes E1-E10: Catalase, RNase A, amylase, proteinase K, lipase II, lysozyme, lipase VII, chymotrypsin VII, trypsin, and chymotrypsin II, respectively. Lanes T1-T10, *Bt*CspB treated by E1-E10. (**b**) The coverage map of *Bt*CspB by LC-MS/MS. The characters with black overlines corresponded to tryptic peptides in Fig. 2d, whereas the characters with red overlines represent the chymotryptic peptides in Fig. 2d. The characters with black, red or blue underlines were the peptides products treated by trypsin, chymotrypsin (II and VII) or proteinase K and detected by LC-MS/MS. The dotted lines show the putative nucleic acid binding motifs (RNP I and RNP II) of the cold shock domain proteins. (**c**) The CD spectra of the proteins. Most of the enzymes used showed strong CD spectra, but α-amylase, lipase VII and lipase II displayed very low background. Thus, the CD spectra of *Bt*CspB treated by α-amylase, lipase VII or lipase II were recorded using CK1 in Table 1 as a control.

To determine the biochemical nature of *Bt*CspB, it was digested with different enzymes and treated at various temperatures and pHs. The bacteriocin activity was completely lost after enzymatic treatment with proteinase K. However, trypsin, RNase A and α-chymotrypsin II did not influence the activity. The residual antibacterial activities after α-chymotrypsin II and α-chymotrypsin VII treatment were 73.9 ± 7.2% and 69.6 ± 2.0%, respectively, using the untreated *Bt*CspB in PBS buffer (CK1) as the CK (Table 1). Herein, the activities of α-chymotrypsin II and α-chymotrypsin VII did not exhibit a significant difference (Table 1). Tricine-SDS-PAGE showed that *Bt*CspB was (partially) digested by trypsin, α-chymotrypsin II, catalase and α-amylase, respectively (Fig. 4a). The digestion results from the treatment with trypsin, chymotrypsin II, chymotrypsin VII and proteinase K were further confirmed by LC-MS/MS (Fig. 4b). The secondary structure composition of the thuricin was further probed using circular dichroism (CD) spectroscopy. Its CD spectra, which had a minimum ellipticity at 200 nm, did not have the representative patterns of α-helical-rich and β-strand-rich proteins or disordered proteins (Fig. 4c). Most of the enzymes in PBS buffer (Table 1) showed strong CD spectra, except for α-amylase, lipase II and lipase VII. Similar to untreated thuricin, α-amylase- or lipase VII-treated thuricin displayed similar CD spectra, with negative bands at 203 nm or 200 nm. However, the lipase II-treated thuricin had negative bands at 227 nm and 207 nm, which is close to the representative pattern of α-helical-rich proteins, which have negative bands at 222 nm and 208 nm.

**Figure 5:**
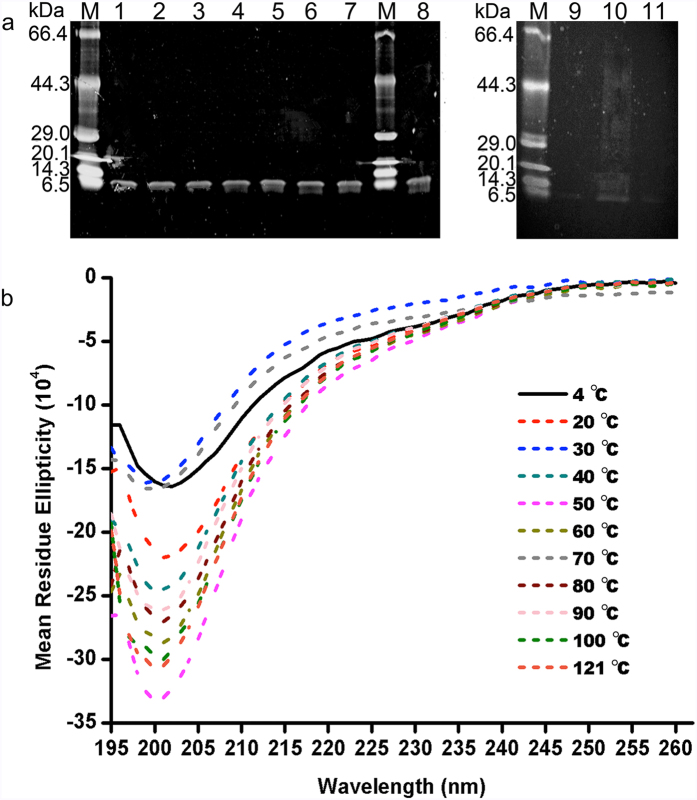
Stability of *Bt*CspB after treatment with different temperatures. (**a**) Tricine-SDS-PAGE. Lane M: TaKaRa premixed protein marker (Broad). Lanes 1–11: samples of *Bt*CspB after treatment at 20 °C, 30 °C, 40 °C, 50 °C, 60 °C, 70 °C, 80 °C, 4 °C (CK), 90 °C, 100 °C and 121 °C. (**b**) The CD spectra of *Bt*CspB treated with different temperatures. The CD spectra of *Bt*CspB treated by different temperatures were obtained after the subtraction of the CD contributed by the corresponding buffer PBS (pH 7.0).

*Bt*CspB was examined for its heat resistance, both as a potential replacement for filter sterilization and as an indication that bacteriocin preparations would survive applications in heated foods. It was stable after treatment at ≤40 °C for 30 min (*P* < 0.05, *P* values in Table 1). After heat treatment at 50 °C or 60 °C for 30 min, 82.8% and 39.8% of the bacteriocin activity remained, respectively. Its activity was completely lost at higher temperatures (Table 1). Tricine-SDS-PAGE showed that the thuricin did not degrade under all conditions (Fig. 5a). Thus, the samples were further investigated by differential scanning calorimetry (DSC) for their thermal transitions and by CD spectroscopy for their secondary structures. The onset temperature (*T*_*o*_), peak temperature (*T*_*p*_, also called melting temperature, *T*_*m*_) and conclusion temperature (*T*_*c*_) were measured to characterize the thermal properties of *Bt*CspB. The results of DSC (Table 2) showed that all samples had the same *T*_*o*_ (*P* > 0.05). When compared with the CK (thuricin treated at 4 °C), thuricin treated at 70 °C had a lower *T*_*p*_ (*T*_*m*_). The difference might be explained by different structural conformations. However, based on the observed CD spectra, all samples had similar minimum ellipticities near 199–202 nm (Fig. 5b). In this context, it is possible that they shared similar structural conformations with minor differences.

**Figure 6:**
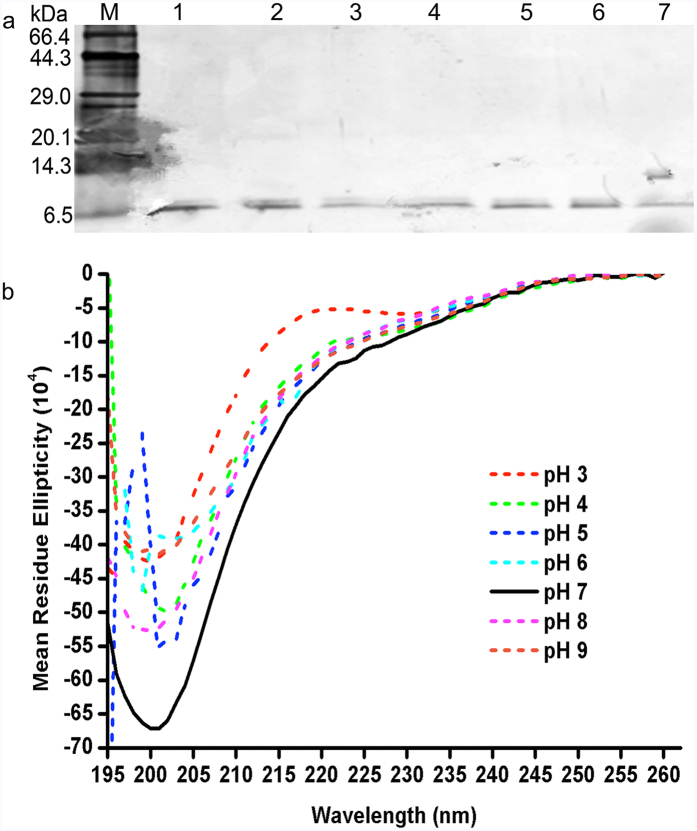
Stability of *Bt*CspB after treatment at different pH values. (**a**) Tricine-SDS-PAGE. Lane M: TaKaRa premixed protein marker (Broad). Lanes 1–7: samples of *Bt*CspB after treated at pH 3–9. (**b**) The CD spectra of pH treated *Bt*CspB. The CD spectra of *Bt*CspB treated by different pH values were obtained after the subtraction of the CD contributed by the corresponding buffers: 50 mM citric acid buffer (pH 3, 4, 5 and 6), phosphate buffer (pH 7) and Tris-HCl buffer (pH 8, 9).

*Bt*CspB was stable at pH 5–7 and had lower activities at pH 3, 4, 8 and 9 (*P* < 0.05, *P* values in Table 1). Tricine-SDS-PAGE indicated that *Bt*CspB was stable at all pH values (Fig. 6a). The results of thermal transitions (Table 2) showed that the pH 6 and pH 7 samples shared the same *T*_*p*_ (*T*_*m*_), whereas the pH 6 treated *Bt*CspB had same *T*_*p*_ (*T*_*m*_) as the pH 4, pH 5 and pH 9 treated samples (*P* > 0.05). Compared with the other samples, the pH 8 treated sample had the highest *T*_*p*_ (*T*_*m*_) (*P* < 0.05). Based on the results of the CK, pH 4, pH 5 and pH 6 induced lower *T*_*o*_ values, whereas pH 8 conferred a higher *T*_*o*_ value (*P* < 0.05). All samples had the same *T*_*c*_as that of the CK (*P* > 0.05). To gain more information about this stability, the CD spectra were determined for all samples. Figure 6 shows that the CD spectra of *Bt*CspB under all conditions tested had negative bands at 195–201 nm, which is indicative of relatively varied secondary structure content.

### Antibacterial spectrum of *Bt*CspB

Among 11 Gram-positive species and 12 Gram-negative species of potential food-borne bacteria (see Supplementary Table 2), 10 μg/mL *Bt*CspB was active against *B. cereus* strains 0938 and ATCC 10987 with MIC values of 3.125 μg/mL and 0.781 μg/mL, respectively. The MBC values of *Bt*CspB for the *B. cereus* strains 0938 and ATCC 10987 were determined to be 12.5 μg/mL and 6.25 μg/mL, respectively. *Bt*CspB did not detectably inhibit any other strains, even at a high concentration (100 μg/mL), instead exhibiting an extremely narrow antibacterial spectrum and high specificity. It did not inhibit its producing strain, which indicated that Bt BRC-ZYR2 is likely to harbor at least one immunity gene. The results also showed that the MICs of *Bt*CspB against five typical Bt strains BRC-LJ2, BRC-LLP29, BRC-HZM3, BRC-CWS1 and BRC-WCB2 (see Supplementary Table 1) isolated from different sources (food, plant, soil, water and feces) are all 100 μg/mL, indicating these strains were not highly sensitive to *Bt*CspB.

To commercially apply *Bt*CspB in food products, it is better to examine the compatibility between *Bt*CspB and major probiotics. The results showed that a high concentration (100 μg/mL) of *Bt*CspB lacked activity against four probiotics, including *Lactobacillus bulgaricus* (GIMI.80), *Lactobacillus acidophilus* (GIMI.208), *Bifidobacterium infantis* (CICC6069) and *Bifidobacterium bifidum* (CICC6071). Based on these results, *Bt*CspB is likely to be compatible with commercial food products that contain these probiotics.

## Discussion

The *Bacillus* species are industrially important for a variety of reasons, including their excellent safety record; their rapid growth rates, which result in short fermentation cycles; and their high capacity for protein secretion into the extracellular medium^17^. Recently, there has been a marked interest in identifying and characterizing extracellularly secreted Bt bacteriocins with a primary interest in expanding their use in both industrial and agronomical avenues^12^. In this study, bacteriocin-producing Bt strains active against *B. cereus* were isolated from milk, jelly, bryophyta, soil and water (see Supplementary Table 1). Of them, Bt BRC-ZYR2 was isolated from uranium-contaminated soil. Bacteriocins produced by various strains of Bt could provide viable alternatives to antimicrobials^5^.

Most Bt strains produce bacteriocins during their stationary phase of growth, coinciding with sporulation and Cry synthesis^5^. However, thuricin CD displayed a narrow spectrum of inhibition, which was restricted to spore-forming *Clostridium difficile*, *Listeria monocytogenes* (Lm), and *Listeria fermentum* during the late logarithmic phase and the stationary phase of growth^18^. Thuricin 7, active against *B. cereus*, Lm and *Streptococcus pyogenes*, was also detected at the end of the logarithmic phase and reached a maximum concentration during the middle stationary phase^19^. Bt BRC-ZYR2 bacteriocins displayed similar kinetics to those for thuricin CD and thuricin 7^18^,^19^. However, the activity of Bt BRC-ZYR2 bacteriocins disappeared at 26 h and reappeared 34 h, which is highly unusual. Further experiments like transcriptome or proteome would help to unravel the miracle. The activity that is observed after 34 h was due to the same substance as that produced earlier in the growth phase (Fig. 1b). In addition, Bt BRC-ZYR2 was active against *B. cereus* at 12 h after incubation in the screening experiment of bacteriocin producers, but it did not show bacteriocin activity at the same time in the kinetics experiment. Such conflicting evidence might be due to the various volumes of TSB medium used: 100 mL per flask was used for the screening experiment, and 200 mL per flask was used for the kinetics experiment.

*Bt*CspB shares a high sequence identity with *Bc*CspB*, Bs*CspB and *Ec*CspB, which are implicated in transcription and translation regulation during cold shock stress^20^,^21^,^22^,^23^,^24^,^25^,^26^. CspB harbors a unique structural feature of all cold-shock proteins (CSPs): a cold shock domain (CSD) containing a five-stranded antiparallel β-barrel. Figure 3 shows that the two putative RNP motifs of CSPs are conserved in CspB proteins^24^.

In addition to response to cold shock stress, CspB can promote efficient Listeriolysin O (LLO) production and, hence, the virulence responses of Lm^27^. It is important for NaCl, pH and ethanol stress responses and for the motility of *Clostridium botulinum* ATCC 3502^28^. In addition, DroughtGard^TM^ maize was developed through the constitutive expression of *Bs*CspB to improve the performance of maize (*Zea mays*) under limited water conditions^29^. The newly discovered bacteriocin activity of *Bt*CspB definitely confers a novel role to CspB. Undoubtedly, the causes of the bacteriocin activity of a putative cold shock protein are of interest, but they remain unknown. Therefore, the 3D structure of the *Bt*CspB with motifs or domains responsible for the novel bacteriocin activity needs to be solved. The transcription and translation regulation of *Bt*CspB on the transcriptome and proteome of *B. cereus* would also be of helpful to unravel its mode of action as a bacteriocin.

*Bt*CspB completely lost its antibacterial activity after enzymatic treatment with proteinase K (Table 1), showing its proteinaceous nature^30^. Based on the data of MALDI-TOF MS (Fig. 2c), N-terminal amino acid sequencing (Fig. 2d, also see Supplementary Fig. 4), LC-MS/MS (Fig. 2d, also see Supplementary Fig. 5), and the strain’s draft genome sequence, *Bt*CspB was not glycosylated and did not carry lipid anchors. The α-amylase-treated thuricin displayed a similar CD spectrum with that of the untreated thuricin, which did not have any representative patterns of α-helical-rich and β-strand-rich proteins or disordered proteins. The lipase II-treated thuricin, however, had a representative pattern of α-helical-rich proteins (Fig. 4c)^28^. Herein, lipase II-dependent change in the structure of *Bt*CspB is likely to render a partial loss of its antimicrobial effect, while the mode of action of α-amylase on the thuricin remained unknown^31^. Overall, 68.9% of its activity was retained after treatment with lysozyme, which is a negative effector in the oral cavity and intestinal tract, thus displaying promise for the use of *Bt*CspB in food preservation and treatment of *B. cereus*-associated diseases^30^. If crude antimicrobial compounds were used, the finding of partial loss activity after catalase treatment indicated that there was hydrogen peroxide in the compounds^32^. It was found that the concentration of thuricin *Bt*CspB is decreased after catalase treatment (Lane T1 in Fig. 4a). Herein, the partial loss activity after the catalase treatment was likely due to the partial degradation of thuricin *Bt*CspB. The degradation of *Bt*CspB by trypsin, chymotrypsin II, chymotrypsin VII and proteinase K were confirmed by tricine-SDS-PAGE (Fig. 4a) and LC-MS/MS (Fig. 4b).

As far as temperature stability was concerned, *Bt*CspB retained its full activity against *B. cereus* when treated for 30 min at ≤40 °C (Table 1). Tricine-SDS-PAGE showed that *Bt*CspB was intact after treatment at different temperatures (Fig. 5a). Its moderately heat stable nature might have been due to a modest increase in the temperature, which resulted in its unfolding and the loss of secondary and tertiary structure of the protein^33^. The CD results, however, displayed similar secondary structures (Fig. 5b). Compared with the CK, *Bt*CspB exposed to 70 °C had a lower *T*_*m*_ (Table 2). These results indicated that the loss of activity was not due to degradation or a large conformational change.

The chemical and physical properties of a food, e.g., pH, salt and fat content, may have a significant role in the suitability of a particular bacteriocin^34^. Most Bt bacteriocins are stable under acidic and alkaline conditions and are thermotolerant^5^. Similarly, *Bt*CspB was active over broad ranges of pH from 3 to 9. Since *Bt*CspB has 11 negatively charged residues (Asp + Glu) and 7 positively charged residues (Arg + Lys) with a calculated isoelectric point of 4.84, a possible explanation for such phenomenon is that the net charge of the bacteriocin is also involved in its adsorption to the surface of the cells’ Gram-positive bacteria. In general, bacteriocins do not adsorb well to bacterial cells at pH 5 or lower, compared to their activities at physiological pHs 11–13. At pH 6 or above, bacteriocin molecules adsorb to the surface of bacteriocin-producing cells and other Gram-positive bacteria^35^. All samples remained intact after treatments at different pHs, as detected by tricine-SDS-PAGE (Fig. 6a). It is likely that all samples shared a similar secondary structure (Fig. 6b). The pH 6 and pH 7 samples had the same *T*_*p*_ (*T*_*m*_), whereas the pH 6 treated sample shared the same *T*_*p*_ (*T*_*m*_) with the pH 4, pH 5 and pH 9 treated samples (Table 2). The pH 8 treated sample had the highest *T*_*p*_ (*T*_*m*_) (Table 2).

Although some bacteriocins from Bt strains (Entomocin 420, Kurstacin 287, Kenyacin 404, Morricin 269 and Tolworthcin 524) present a wide antibacterial spectrum^5^,^36^, thuricin 439 and thuricin CD were good examples of Bt bacteriocins with a narrow spectrum^5^,^37^. For example, thuricin 439 only affects the growth of *B. cereus*, Bt and *Listeria innocua* 4202^37^. In this study, *in vitro* tests at a neutral pH were performed using 23 pathogenic species and *Bt*CspB. The strains (0938 and ATCC 10987) of opportunistic pathogen *B. cereus,* a common cause of food poisoning, were found to be very highly sensitive, with MIC values of 3.125 μg/mL and 0.781 μg/mL and MBC values of 12.5 μg/mL and 6.25 μg/mL, respectively. *B. cereus* is widespread in nature and frequently isolated from soil and growing plants, but it is also well adapted for growth in the intestinal tract of insects and mammals to cause an emetic or a diarrhoeal type of food-associated illness^38^. Because of its outstanding ability to adhere to stainless steel surfaces of dairy plant and form biofilm, *B. cereus* can lead to serious hygiene problems and economic loss due to spoilage of dairy products and equipment impairment^39^. The results also showed that *Bt*CspB was not toxic to *Lactobacillus* and *Bifidobacterium*, which are available in commercial probiotic-containing food products^40^. Collectively, the incorporation of *Bt*CspB bacteriocin into foods has the potential to prevent the growth of spoilage bacteria to extend food storage life and control food-borne diseases.

Bacteriocins can have a broad (effective against multiple genera) or narrow (effective against specific species) spectrum of activity. The diversity offers the possibility of multiple applications in the food and pharmaceutical industries^41^. Although several broad-spectrum bacteriocins exist that can be used to target infections of unknown aetiology, potent narrow-spectrum bacteriocins, including thuricin CD targeting *Clostridium difficile*, have also been identified that can control targeted pathogens without negatively affecting commensal populations^42^,^43^. One way to address novel targets and to improve the efficacy and stability of relative narrow-spectrum bacteriocins is through their manipulation by engineering and generating more effective bacteriocins based on rational design. Some engineered bacteriocins include microcin B17, geobacillin I, nisin, lacticin 3147, enterocin E50–52 and pediocin PA-1^44^. Bacteriocins can even be produced *in situ* in the gut by probiotic bacteria to combat intestinal infections^42^. Another method is to synergize bacteriocin in combination with other bacteriocins and conventional antibiotics. For example, poly-l-lysine and nisin A act synergistically against *B. cereus* and *L.* monocytogenes^44^.

In summary, *Bt*CspB, a 7366.1 Da peptide produced by Bt BRC-ZYR2, is active against *B. cereus* 0938 and ATCC 10987. *Bt*CspB has a high similarity with the cold shock protein CspBs. It is heat resistant up to 60 °C. Because of this, it could be used in pasteurized foods. Its stability over a wide pH range indicates that it could be used in acidic and non-acidic foods. Its proteinaceous nature also renders it safe for human consumption. These properties make *Bt*CspB a promising agent in food preservation and for treatment against *B. cereus*-associated diseases. Since research in this area is still in its infancy, a thorough investigation on the mode of action of *Bt*CspB as a bacteriocin, bacteriocin engineering and bacteriocin synergy is of considerable interest to unravel the roles of *Bt*CspB and develop novel *Bt*CspB bacteriocins with enhanced efficacy for its potential applications.

## Methods

### Strains, media and growth conditions

The food-borne bacteria and Bt used for the antagonist assay and their culture conditions are listed in Supplementary Tables 1 and 2. For the compatibility test between the bacteriocins and four probiotics, *L. bulgaricus* GIMI.80, *L. acidophilus* GIMI.208, *B. infantis* CICC6069 and *B. bifidum* CICC6071, were used as indicator strains. The strains were cultured in Tryptic Soy Broth (TSB) at 30 °C with shaking (150 rpm).

### Screening of Bt bacteriocin producers active against *B. cereus*

The antibacterial activity of Bt strains (see Supplementary Table 1) was determined by an agar well diffusion assay with some modifications^45^. Briefly, 15 mL of TSB with soft agar (0.7%) was mixed with 50 μL (1 × 10^9^ cells/mL) of the indicator strains of *Bacillus cereus* 0938 and ATCC10987 and plated. Then, 1% of the overnight cultures were transferred into TSB (100 mL) for growth at 30 °C for 12 h in a shaker at 150 rpm, and the concentration of the cells was adjusted to 1 × 10^9^ cells/mL. The supernatants were adjusted to pH 6.8 and filtered through a 0.22 μm filter. Then, 50 μL of the cell-free supernatants was added to a 7-mm well in a plate and evaluated for their antibacterial activities.

### Kinetics of bacteriocin production

Bt BRC-ZYR2 was cultured in TSB (200 mL) at 30 °C in a shaker at 150 rpm, and duplicate samples were taken at 2-h intervals over 72 h. One of the samples was used to monitor cell growth spectrophotometrically at 600 nm. The other sample was centrifuged, and the supernatants were adjusted to pH 6.8 and filtered through a 0.22 μm filter. They were then used to evaluate the bacteriocin activity via the modified well-diffusion method as described above^45^,^46^. To determine the kinetics of its production, tricine-SDS-PAGE was used as previously described^47^. The activity measurements were carried out in triplicate, and the average activity measurement was recorded^48^. One unit (U) is defined as 1 mm^2^ of the zone of inhibition. as determined by the well-diffusion method^45^.

### Direct detection of bacteriocin activity in gel-overlay assays

To estimate the molecular weight (MW) of the purified bacteriocins, tricine-SDS-PAGE was performed as previously described^47^. After electrophoresis, the gel was cut vertically. The first part containing the purified bacteriocins and a protein marker were stained with Coomassie brilliant blue (CBB) R-250 to estimate the MW. The second part containing the purified bacteriocins was assayed for the direct detection of bacteriocin activity. The latter was fixed in isopropanol (25%) and acetic acid (10%) for 30 min, washed with dH_2_O for 3 h, and overlaid in a Petri dish of soft TSA medium (5 mL) containing the indicator strain *B. cereus* 0938. The Petri dish was incubated at 30 °C for 24 h. The direct detection of bacteriocin was observed by the presence of an inhibition zone^12^. The pictures of two parts were taken by a PC1266 camera (Cannon Inc., Tokyo, Japan) and cropped with Adobe Photoshop 7.0.1 (Adobe Systems Incorporated, San Jose, CA, USA).

### Bacteriocin purification

The bacteriocin was purified to homogeneity by ammonium sulfate precipitation, Sephadex G-50 column chromatography and two rounds of DEAE-52 column chromatography. Briefly, Bt BRC-ZYR2 was cultivated in TSB (200 mL) until the highest bacteriocin activity was detected in the kinetics of the bacteriocin production. The cell-free supernatants were concentrated with saturation ammonium sulfate (100%) at 4 °C with constant stirring overnight. The precipitated proteins were centrifuged at 16,000 g for 30 min at 4 °C and dissolved in 10 mM Tris-Cl buffer, pH 7.4^45^. Then, 4 mL of the partially purified bacteriocins, filtered through a 0.22 μm filter, was applied to a Sephadex G-50 column (10 mm × 600 mm) equilibrated with 10 mM Tris-Cl, pH 7.4^12^. The samples were eluted at 1 mL/min with 10 mM Tris-Cl, pH 7.4 for 190 min. The elution was detected with a N2000 Chromatography Workstation (Zhejiang University, China), and the bacteriocin activity was determined using the agar well-diffusion assay. The active fractions were then pooled and dialyzed overnight. After the DEAE-52 cellulose column (16 mm × 300 mm) was equilibrated with 10 mM Tris-Cl, pH 7.4, 4 mL of the above sample was further purified using 10 mM Tris-Cl, pH 7.4 containing NaCl (0 M, 0.1 M or 0.6 M) at a flow rate of 1 mL/min. The active fractions were then pooled and dialyzed overnight. Then, a second round of DEAE-52 column chromatography was performed and eluted with 10 mM Tris-Cl, pH 7.4 containing NaCl (0 M, 0.16 M, 0.3 M or 0.6 M). A pure BRC-ZYR2 bacterioncin (designated thuricin *Bt*CspB) that was found to be active against *B. cereus* 0938 was pooled and dialyzed overnight against 10 mM Tris-Cl, pH 7.4.

### Determination of the primary structure of *Bt*CspB

The molecular mass of *Bt*CspB was determined using the AB SCIEX TOF/TOF™ 5800 System (AB SCIEX, Foster City, California, USA) at the Shanghai Institute of Biochemistry and Cell Biology, Chinese Academy of Sciences (Shanghai, China). The N-terminal amino acid sequencing was performed using an Edman degradation with a PPSQ-33A protein sequencer (Shimadzu Co., Ltd, Kyoto, Japan) at the Shanghai Institute of Biochemistry and Cell Biology, Chinese Academy of Sciences (Shanghai, China). The draft genome sequence of Bt BRC-ZYR2 was obtained using a Roche/454 GS FLX sequencer (454 Life Sciences, Branford, Connecticut, USA) by the Insect-Resistant Biotechnology Laboratory, Plant Protection Institute, Chinese Academy of Agricultural Sciences (Beijing, China).

*Bt*CspB was further identified using LC-MS/MS. Briefly, 10 μg of the sample was solubilized in UA buffer (8 M Urea, 150 mM Tris-HCl, pH 8.0), vortexed, and sonicated for 2 min. Then, 5 μL of 200 mM DTT reducing reagent was added to the sample and gently vortexed. The mixture was left at room temperature for 90 min. Then, 20 μL of 200 mM iodoactamide alkylating reagent was added. The sample was gently vortexed, and the mixture was incubated at room temperature for 1 h in dark. After 300 μL of 25 mM NH_4_HCO_3_ was added to reduce the urea concentration, trypsin (or chymotrypsin) solution was added to a final ratio of 1:50–1:10, respectively (w/w, protease:*Bt*CspB), gently vortexed, and incubated at 37 °C for 16–20 h. *Bt*CspB was desalted using C18 Cartridges (Empore™ SPE Cartridges C18 (standard density), bed I.D. 7 mm, volume 3 mL, Sigma-Aldrich, USA), concentrated by vacuum centrifugation, and reconstituted in 40 μL of 0.1% (v/v) formic acid. LC-MS/MS **e**xperiments were performed on a Q Exactive mass spectrometer that was coupled to an Easy nLC (Thermo Fisher Scientific, Waltham, Massachusetts, USA). The peptide mixture was loaded onto a C18-reversed phase column (15 cm long, 75 μm inner diameter) packed in-house with RP-C18 5 μm resin in buffer A (0.1% formic acid in HPLC-grade water) and separated with a linear gradient of buffer B (0.1% formic acid in 84% acetonitrile) at a flow rate of 250 nL/min controlled by IntelliFlow technology. The MS data were acquired using a data-dependent top10 method, which dynamically chose the most abundant precursor ions from the survey scan (300–1800 m/z) for HCD fragmentation. The determination of the target value is based on the predictive Automatic Gain Control (pAGC). The dynamic exclusion duration was 20 s. The survey scans were acquired at a resolution of 70,000 at m/z 200, and the resolution for the HCD spectra was set to 17,500 at m/z 200. The normalized collision energy was 27 eV, and the underfill ratio, which specifies the minimum percentage of the target value likely to be reached at maximum fill time, was defined as 0.1%. The instrument was run with the peptide recognition mode enabled. The MS/MS spectra were searched using the MASCOT engine (Matrix Science, London, UK; version 2.2) against the *Bacillus thuringiensis* sequence database (downloaded at August 9, 2015). For protein identification, the following options were used: peptide mass tolerance = 20 ppm, MS/MS tolerance = 0.1 Da, enzyme = trypsin/chymotrypsin, missed cleavage = 2, fixed modification: carbamidomethyl (C), variable modification: oxidation(M), ion score >20 and FDR < 0.01 at the peptide and protein level.

The *Bt*CspB gene was amplified with a pair of PCR primers (CB1: ATGCAAAACGGTAAAGTAAAAT; CB2: TTACTTCTTGTTTACGTTAGTAGCT). The PCR product was cloned by recombinant DNA techniques and sequenced by a Model 3730 gene sequencer from Applied Biosystems (Foster City, California).

### Sequence-based analysis of *Bt*CspB

The molecular weight, isoelectric point and amino acid composition profile were computed by ExPaSy ProtParam (http://web.expasy.org/protparam/). A homology search was performed using BLAST (Basic Local Alignment Search Tool, NCBI-algorithm BLASTP). The multiple sequence alignment was created by the ClustalW2 program (http://www.ebi.ac.uk/Tools/msa/clustalw2/).

### Sensitivity of *Bt*CspB to enzymes, temperature and pH

The sensitivity of *Bt*CspB to enzymatic proteolysis was tested by treatment with α-amylase (Sigma-Aldrich), lipase VII (Sigma-Aldrich), lipase II (Sigma-Aldrich), RNase A (Sigma-Aldrich), lysozyme (Sigma-Aldrich), catalase (Sigma-Aldrich), trypsin (Sigma-Aldrich), proteinase K (Sigma-Aldrich), α-chymotrypsin-VII (Sigma-Aldrich) or α-chymotrypsin II (Sigma-Aldrich) at a final concentration of 1 mg/mL each. The untreated bacteriocin in PBS buffer (CK1), PBS buffer alone (CK2) and each enzyme in PBS buffer (CK3) were used as controls. The reactions were incubated at 37 °C for 2 h at pH 7.0^45^.

The thermal sensitivity of *Bt*CspB in PBS buffer (pH 7.0) was evaluated at different temperatures (4, 20, 30, 40, 50, 60, 70, 80, 90 and 100 °C) for 30 min and in an autoclave at 121 °C for 30 min^45^.

The pH stability of *Bt*CspB was estimated after 2 h at 4 °C in the following buffers (50 mM): citric acid buffer (pH 3, 4, 5 and 6), PBS buffer (pH 7) and Tris-HCl buffer (pH 8, 9). *Bt*CspB dissolved in phosphate buffer (pH 7.0) was used as a control (CK).

The above residual antibacterial activities were calculated using the modified well-diffusion assay with *B. cereus* 0938 as the indicator strain, and the related buffers were used as negative controls. The experiments were performed in triplicate (*n* = 18). The Tamhane’s T2 test in the SPSS software version 13.0 (SPSS Inc., Chicago, IL, USA) was used for the analysis. Alpha levels of 0.05 were used for the statistical test.

To provide information on its susceptibility to degradation, tricine-SDS-PAGE was performed for the samples treated in different ways as previously described^47^. When degradation occurred, the samples, which were treated with trypsin, chymotrypsin II, chymotrypsin VII and proteinase K, were subjected to the LC-MS-MS experiments described as the above.

The thermal transitions of *Bt*CspB treated with different temperatures and pH values were investigated with the use of differential scanning calorimetry (STA 449 F3 Jupiter^®^, NETZSCH Vakumix GmbH, Weyhe–Dreye, Germany). A sample (94–4,113 μg) was added to a DSC pan. The pan was sealed, equilibrated for 30 min at room temperature, and then heated from 25 to 100 °C at the rate of 10 °C/min. The onset temperature (*T*_*o*_), peak temperature (*T*_*p*_; also called the melting temperature, *T*_*m*_) and conclusion temperature (*T*_*c*_) were measured to characterize the thermal properties of *Bt*CspB. The experiments were performed in triplicate (*n* = 3). The Tamhane’s T2 test or least-significant difference (LSD) test found in SPSS software version 13.0 (Chicago, USA) were used in the analyses. Alpha levels of 0.05 were used for the statistical test.

To probe the secondary structures^49^, the CD spectra of the above samples and their solvents with or without enzymes (Table 1) were recorded using a Chirascan^TM^ circular dichroism spectrophotometer (Applied Photophysics, Leatherhead, Surrey, UK) at 37 °C using a 1 mm pathlength quartz cell using over a measurement range of 195–260 cm at a scanning speed of 1 nm/s, a bandwidth of 1 nm, and 1 s per point. The spectra were averaged over three consecutive scans, followed by the subtraction of the CD signal of the solvent with or without enzymes (Table 1). The peptide concentrations for the CD measurements were 0.27–2.04 mM.

### Antibacterial spectrum of *Bt*CspB

*Bt*CspB was used to determine the antibacterial spectrum against the indicator strains (see Supplementary Table 2), Bt BRC-ZYR2 and four probiotics using the agar well-diffusion assay. TSB and Milli-Q water were used as negative controls.

The MIC of *Bt*CspB was determined using a microplate dilution method in Mueller-Hinton broth (MH, Becton, Dickinson & Co., Sparks, MD). TSB was used as the negative controls for the growth. *B. cereus* 0938 and ATCC 10987 alone were used as the positive controls of growth and the indicator strains. To test the toxicity of 0.2–100 μg/mL *Bt*CspB against other Bt strains, five typical Bt strains BRC-LJ2, BRC-LLP29, BRC-HZM3, BRC-CWS1 and BRC-WCB2 (see Supplementary Table 1) isolated from different sources (food, plant, soil, water and feces) were chose as indicator strains. Briefly, bacteria were grown at 37 °C in TSB, harvested while in the exponential phase (OD600 nm: 0.6–0.8), and centrifuged (8 × 10^3^ g for 10 min). They were then washed with saline (0.15 M NaCl), resuspended in Muller Hinton (MH) broth at the concentration of approximately 2 × 10^6^ CFU/mL, and then distributed in triplicate into 96-well plates (50** **μL/well). The cells were mixed with increasing concentrations of the antimicrobial peptides dissolved in MH broth (final conc. 0.2–100 μg/mL, 50 μL/well) at 4 °C for 12 h and incubated at 37 °C for 20 h. The MIC is defined as the minimal peptide concentration at which 100% inhibition of the microbial growth was observed, and the value is determined by measuring the absorbance at 540 nm^50^,^51^. Three independent experiments were performed to ensure reproducibility.

A plate count of viable cells was performed to evaluate the minimum bactericidal concentration (MBC) of *Bt*CspB against the *B. cereus* strains. Briefly, 10 μL of bacterial solutions from each well that defined the MIC and the wells with concentrations above the MIC were spread onto MH agar plates. The bacterial colonies were counted after the plates were incubated at 37 °C for 24 h. The MBC was defined as the lowest concentration of an antibiotic that killed 99.9% of the total bacteria. Three independent experiments were performed to ensure reproducibility^51^.

## Additional Information

**How to cite this article**: Huang, T. *et al.* Purification and Characterization of a Novel Cold Shock Protein-Like Bacteriocin Synthesized by *Bacillus thuringiensis.* *Sci. Rep.* **6**, 35560; doi: 10.1038/srep35560 (2016).

## Supplementary Material

Supplementary Information

## Figures and Tables

**Table 1:** Effects of enzyme, temperature, and pH on the antibacterial activity of *Bt*CspB.

Treatment		Residual antibacterial activity (% ± SE)	P value
Enzyme			
	CK1 (CK)	100.0 ± 6.5a	N/A
	trypsin	74.3 ± 2.4ab	0.068
	RNase A	74.3 ± 2.1ab	0.062
	α-chymotrypsin II	73.9 ± 7.2ab	0.454
	lipase II	72.1 ± 2.3b	0.03
	α-chymotrypsin VII	69.6 ± 2.0b	0.013
	lysozyme	68.9 ± 4.4b	0.023
	lipase VII	67.7 ± 3.3b	0.009
	catalase	67.4 ± 3.1b	0.007
	α-amylase	67.0 ± 2.6b	0.006
	proteinase K	0.0 ± 0.0c	0.000
Temperature			
	4 °C, 30 min (CK)	100.0 ± 3.0a	N/A
	20 °C, 30 min	91.4 ± 2.3ab	0.810
	30 °C, 30 min	95.5 ± 3.5ab	1.000
	40 °C, 30 min	89.4 ± 3.5ab	0.785
	50 °C, 30 min	82.8 ± 2.2b	0.004
	60 °C, 30 min	39.8 ± 2.5c	0.000
	70 °C, 30 min	0.0 ± 0.0d	0.000
	80 °C, 30 min	0.0 ± 0.0d	0.000
	90 °C, 30 min	0.0 ± 0.0d	0.000
	100 °C, 30 min	0.0 ± 0.0d	0.000
	121 °C, 30 min	0.0 ± 0.0d	0.000
pH value			
	3	74.8 ± 2.4c	0.000
	4	89.4 ± 1.7b	0.000
	5	93.4 ± 3.2ab	0.748
	6	105.3 ± 3.6a	0.984
	7 (CK)	100.0 ± 1.2a	N/A
	8	69.3 ± 0.5c	0.000
	9	65.1 ± 0.9d	0.000

Tamhane’s T2 test was used to analyze the data (n = 18). Each treatment was compared with the control (CK). Different lowercase letters in the column of residual antibacterial activity indicate significant differences (*P* < 0.05).

**Table 2:** Thermal transitions of *Bt*CspB treated at different temperatures and pH values.

Sample	*T*_o_ (°C ± SE)	*T*_p_ (°C ± SE)	*T*_c_ (°C ± SE)
4 °C, 30 min (CK)	57.5 ± 1.1a	61.4 ± 0.1a	63.2 ± 0.3b
20 °C, 30 min	57.5 ± 0.2a	61.6 ± 0.2ab	62.9 ± 0.3b
30 °C, 30 min	56.5 ± 0.2a	61.1 ± 0.1ab	62.8 ± 0.1b
40 °C, 30 min	52.6 ± 2.4a	61.2 ± 0.0ab	63.5 ± 0.4b
50 °C, 30 min	53.1 ± 0.7a	61.4 ± 0.1a	63.4 ± 0.5b
60 °C, 30 min	52.3 ± 2.1a	61.2 ± 0.1a	63.2 ± 0.2b
70 °C, 30 min	56.9 ± 0.4a	59.5 ± 0.1b	62.4 ± 0.3b
80 °C, 30 min	56.1 ± 1.3a	61.1 ± 0.1a	63.0 ± 0.2b
90 °C, 30 min	53.5 ± 1.7a	61.5 ± 0.1a	63.5 ± 0.6b
100 °C, 30 min	49.1 ± 2.0a	59.8 ± 0.3ab	62.9 ± 0.4b
121 °C, 30 min	59.6 ± 0.4a	64.2 ± 0.5ab	66.8 ± 0.6a
pH 3	N/A	N/A	N/A
pH 4	49.5 ± 0.6c	52.5 ± 0.3c	54.3 ± 0.2c
pH 5	49.0 ± 0.1c	53.2 ± 0.4c	61.8 ± 0.0ab
pH 6	48.3 ± 0.4c	54.9 ± 1.9bc	62.4 ± 0.3a
pH 7 (CK)	51.2 ± 1.0b	58.2 ± 0.1b	61.9 ± 0.7abc
pH 8	54.8 ± 0.3a	60.2 ± 0.2a	61.9 ± 0.1ab
pH 9	52.3 ± 0.0b	54.4 ± 0.2c	57.9 ± 0.4bc

*T*_o_, onset temperature; *T*_p_, peak temperature (melting temperature, *T*_m_); *T*_c_, conclusion temperature. The data of pH 3 treatment were too low to be detected by the DSC used. Tamhane’s T2 test was used to analyze the data (n = 3), except for the data of *T*_c_ in the temperature treatment and the data of *T*_o_ in the pH treatment, which were analyzed by LSD test. Different lowercase letters in the column of residual antibacterial activity indicate significant differences (*P* < 0.05).
