# Corrigendum: Purification and Characterization of a Novel Cold Shock Protein-Like Bacteriocin Synthesized by *Bacillus thuringiensis*

**DOI:** 10.1038/srep40975

**Published:** 2017-01-19

**Authors:** Tianpei Huang, Xiaojuan Zhang, Jieru Pan, Xiaoyu Su, Xin Jin, Xiong Guan

Scientific Reports 6: Article number: 35560; 10.1038/srep35560 published online: 10 20 2016; updated: 01 19 2017.

This Article contains an error in the Acknowledgements section:

“We are grateful to professor Zongchao Jia (Queen’s University, Canada) and Dr. Xiaofeng Xie (Fujian Agriculture and Forestry University, China) for their revision of the manuscript; to professor Jie Zhang, Dr. Changlong Shu and Yuanyuan Guo for 454 pyrosequencing of the Bt BRC-ZYR2 genome; to Yi Zhang, Jie Jiang, Baoyu Wang, Xiangyin Luo, Lipeng Lin, Yiying Li, Dongna Ma, Haili Fan, Junmin Yao and Guiping Qian for their technical assistance of bacteriocins; to Xiaoqi Wu and Donglin Wu for their help with DSC; and to Jiangmei Tan and Yang Shi for their assistance with CD”.

should read:

“We are grateful to professor Zongchao Jia (Queen’s University, Canada) and Dr. Xiaofeng Xia (Fujian Agriculture and Forestry University, China) for their revision of the manuscript; to professor Jie Zhang, Dr. Changlong Shu and Yuanyuan Guo for 454 pyrosequencing of the Bt BRC-ZYR2 genome; to Yi Zhang, Jie Jiang, Baoyu Wang, Xiangyin Luo, Lipeng Lin, Yiying Li, Dongna Ma, Haili Fan, Junmin Yao and Guiping Qian for their technical assistance of bacteriocins; to Xiaoqi Wu and Donglin Wu for their help with DSC; and to Jiangmei Tan and Yang Shi for their assistance with CD”.

