# Supplementary material for: Purification and Characterization of a Novel Cold Shock Protein-Like Bacteriocin Synthesized by Bacillus thuringiensis
Source: Sci Rep. 2016 Oct 20;6:35560. doi: 10.1038/srep35560 (PMC5071883; doi:10.1038/srep35560)
Supplement: Supplementary Information [file srep35560-s1.doc]

Supplementary Information

**Purification and Characterization of a Novel Cold Shock Protein-Like Bacteriocin Synthesized by *Bacillus thuringiensis***

Tianpei Huang,^1,2^ Xiaojuan Zhang,^1^ Jieru Pan,^3^ Xiaoyu Su,^1^ Xin Jin,^1^ & Xiong Guan^1,2,*^

^1^State Key Laboratory of Ecological Pest Control for Fujian and Taiwan Crops & Key Laboratory of Biopesticide and Chemical Biology (Ministry of Education), College of Life Sciences, Fujian Agriculture and Forestry University, Fuzhou 350002, Fujian, China. ^2^Fujian-Taiwan Joint Center for Ecological Control of Crop Pests, Fuzhou 350002, Fujian, China. ^3^Fuzhou Center for Disease Control and Prevention, Fuzhou 350004, Fujian, China. Correspondence and requests for materials should be addressed to X.G. ([guanxfafu@126.com](mailto:guanxfafu@126.com))


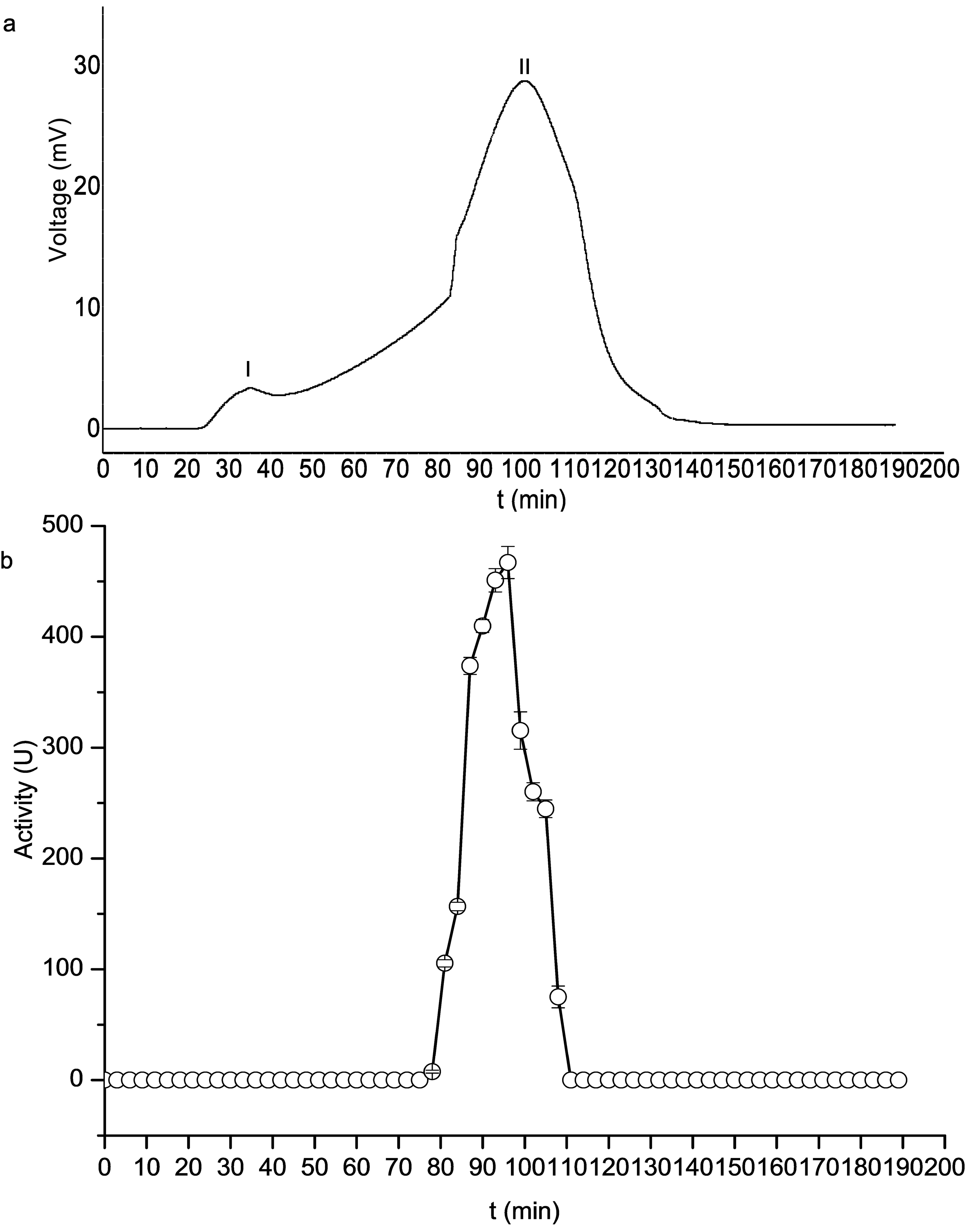


**Supplementary Figure 1. Elution profile of gel filtration chromatography on Sephadex G-50 of Bt BRC-ZYR2 bacteriocins precipitated by 100% ammonium sulfate (a) and detection of their bacteriocin activities (b).** Standard deviations were smaller than the size symbols used in most points.


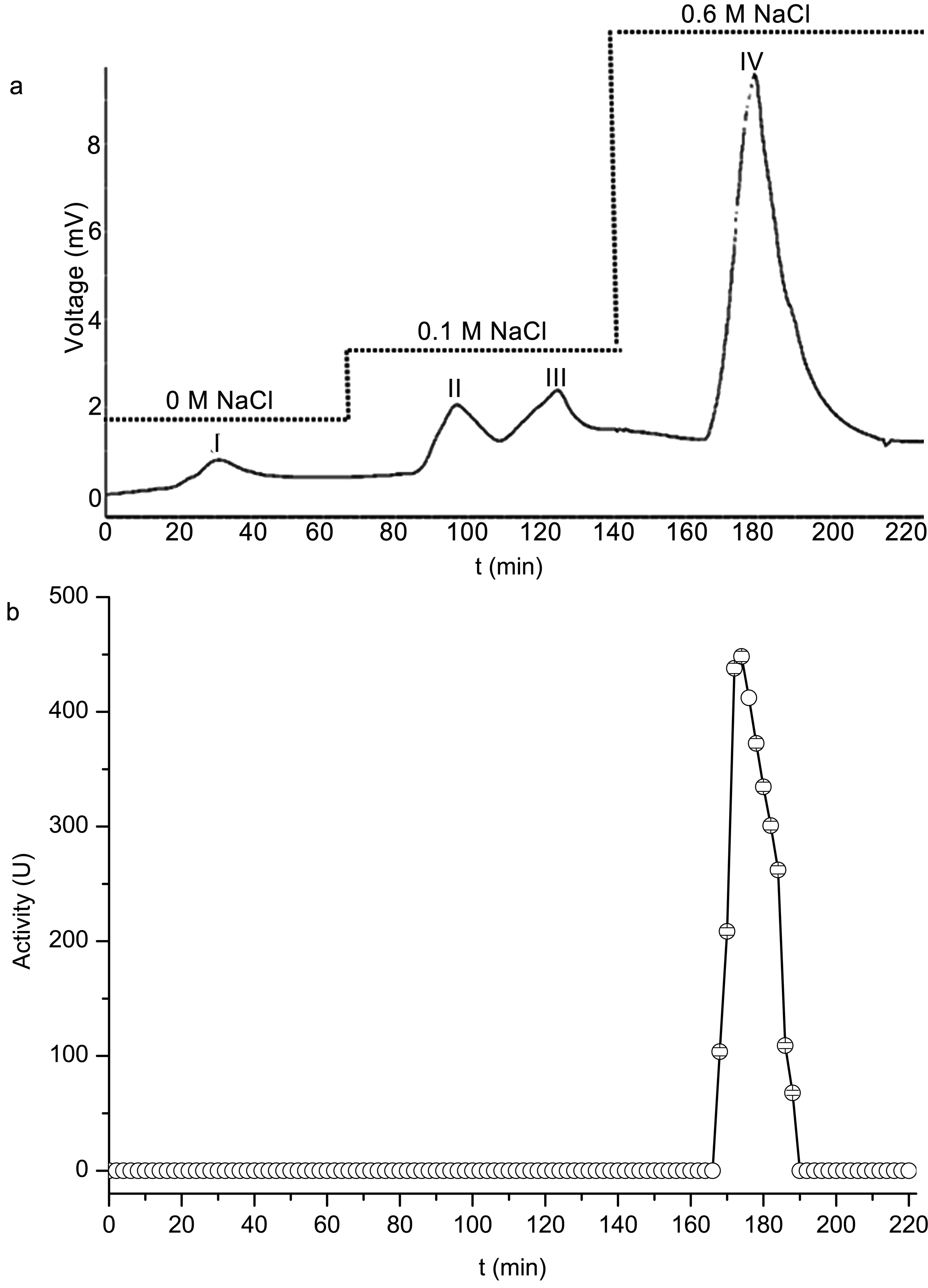


**Supplementary Figure 2. Elution profile of the antibacterial fraction of Bt BRC-ZYR2 bacteriocins after gel filtration chromatography on Sephadex G-50 by first round of cellulose DEAE-52 anion-exchange chromatography (a) and detection of their bacteriocin activities (b).** Standard deviations were smaller than the size symbols used in most points.


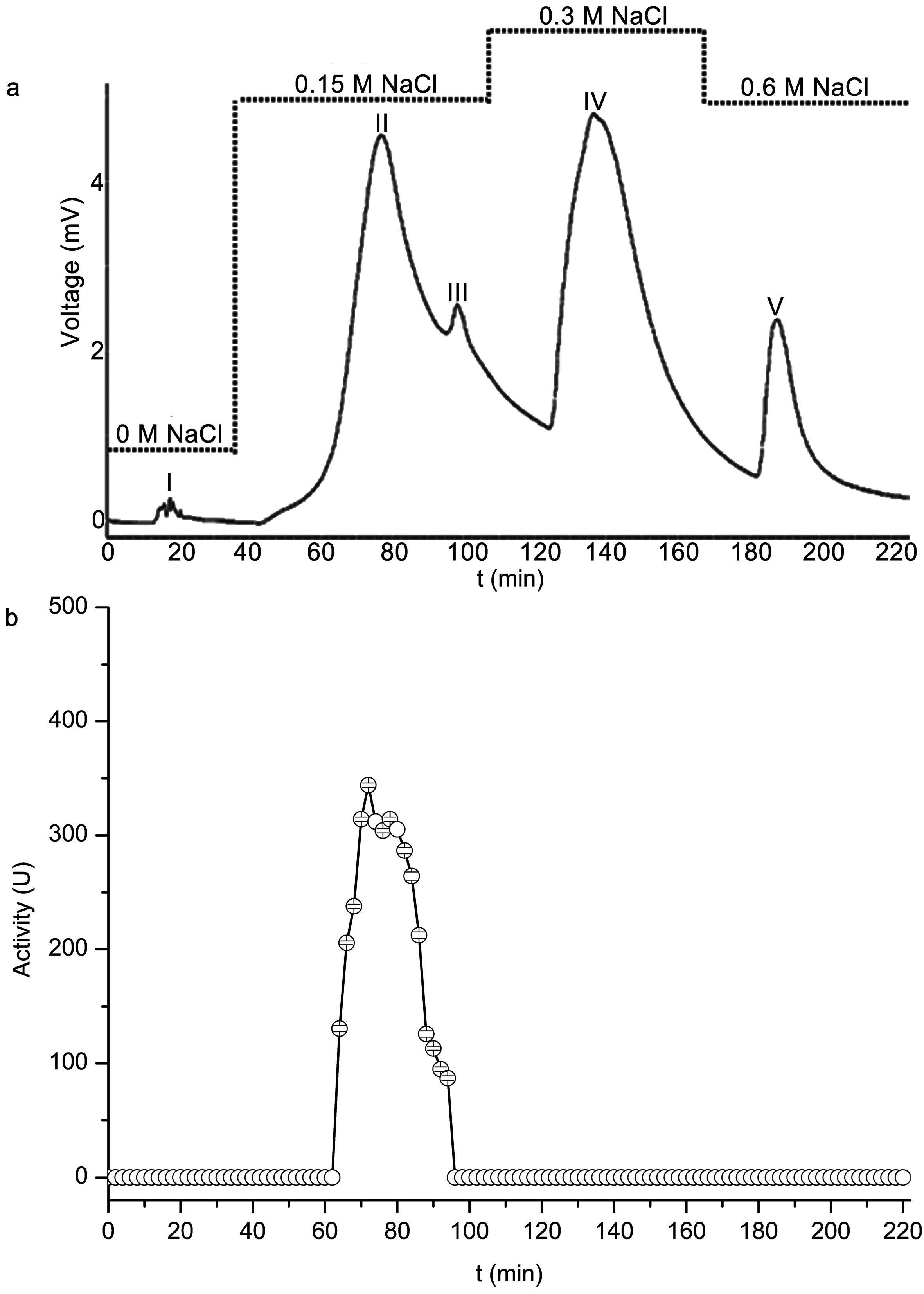


**Supplementary Figure 3.** **Elution profile of the antibacterial fraction of BRC-ZYR2 bacteriocins by second round of cellulose DEAE-52 anion-exchange chromatography (a) and detection of their bacteriocin activities (b).** Standard deviations were smaller than the size symbols used in most points.


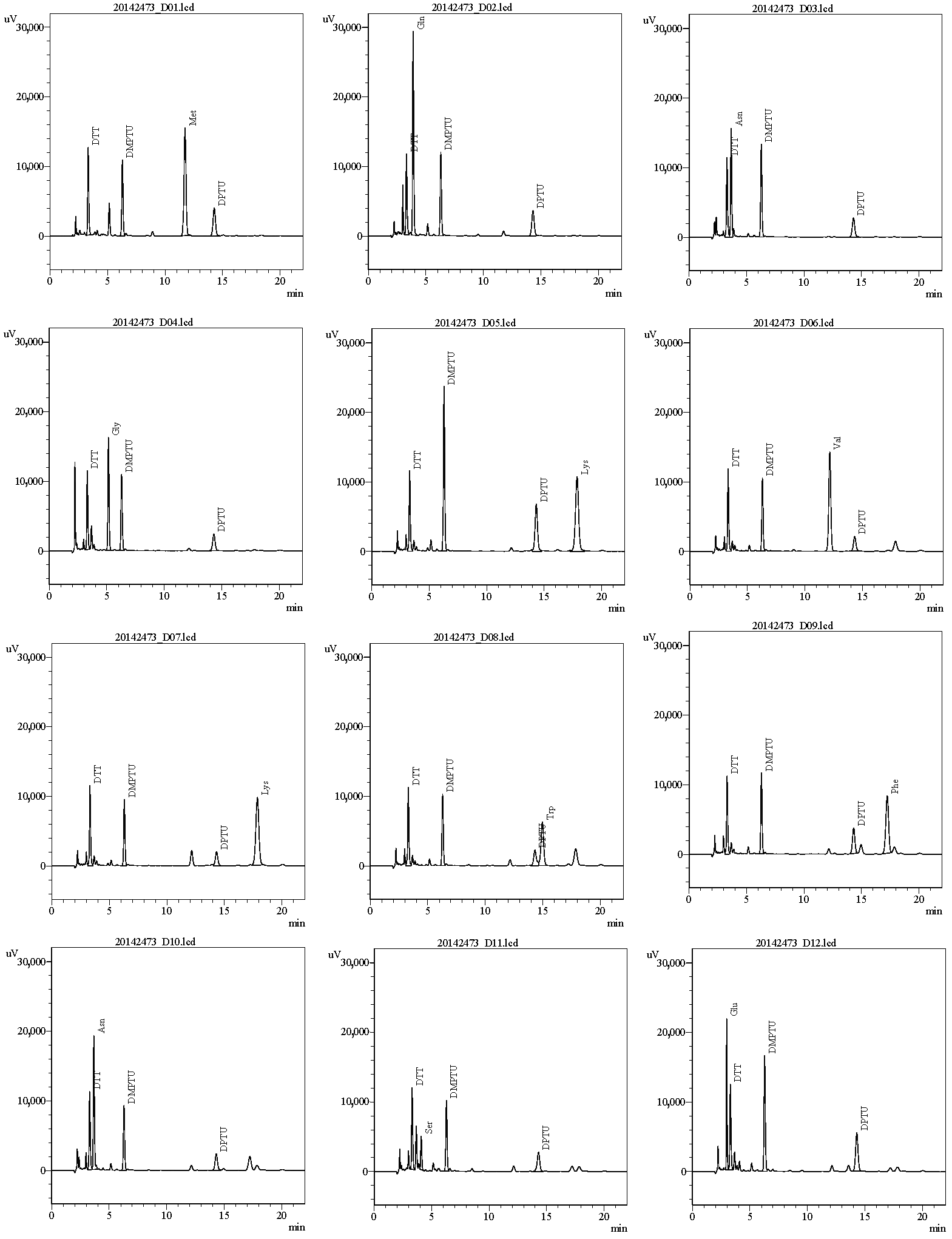


**Supplementary Figure 4. N-terminal amino acid sequencing of thuricin *Bt*CspB performed by Edman degradation.** The first 12 amino acids were determined from Thuricin *Bt*CspB purified with the second gel filtration on cellulose DEAE-52 anion-exchange chromatography.


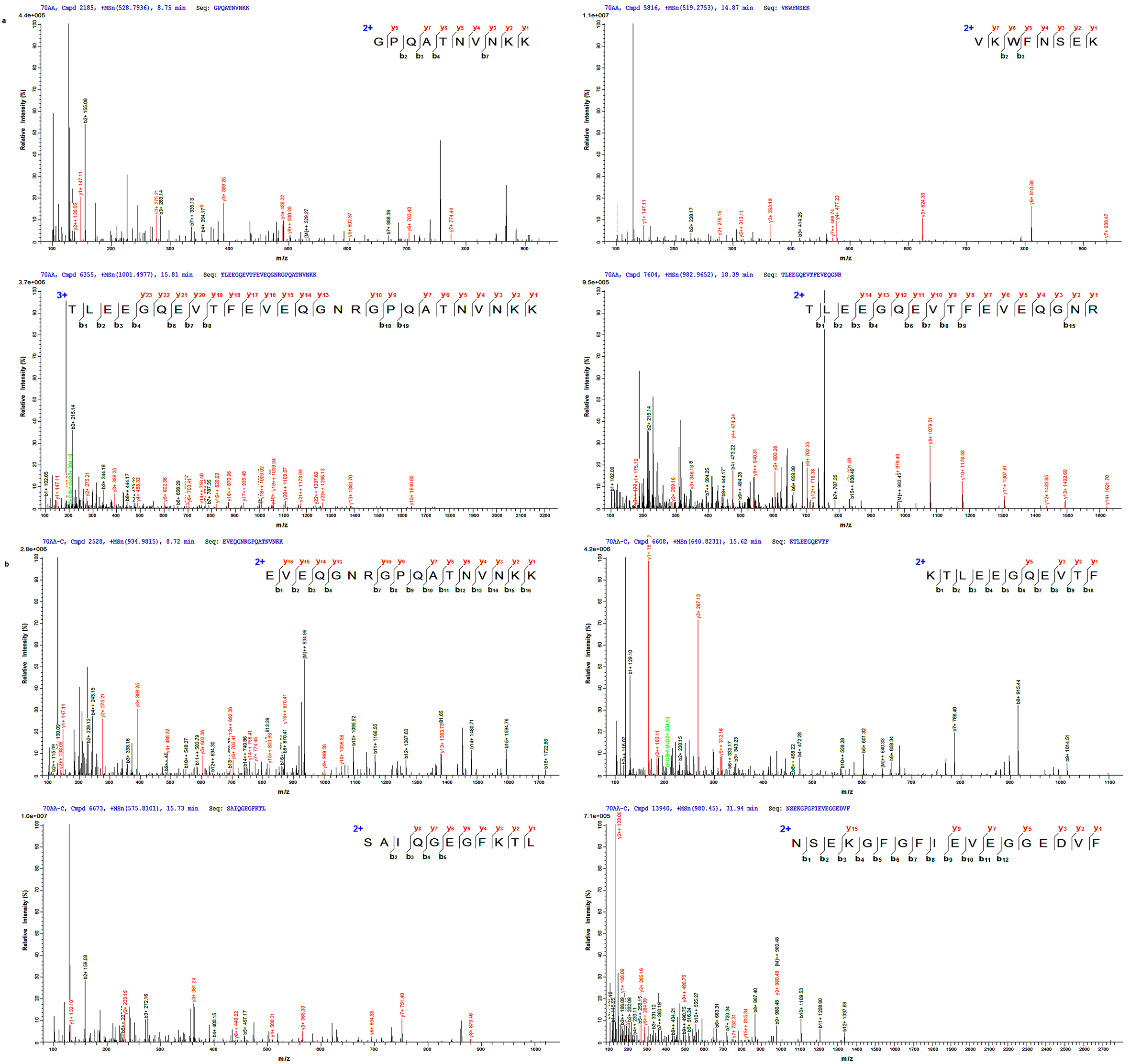


**Supplementary Figure 5. Determination of the primary structure of thuricin *Bt*CspB by LC-MS/MS.** (a) Mass spectrum base peak of four unique peptides of thuricin *Bt*CspB digested with trypsin; (b) Mass spectrum base peak of four unique peptides of thuricin *Bt*CspB digested with chymotrypsin.

| **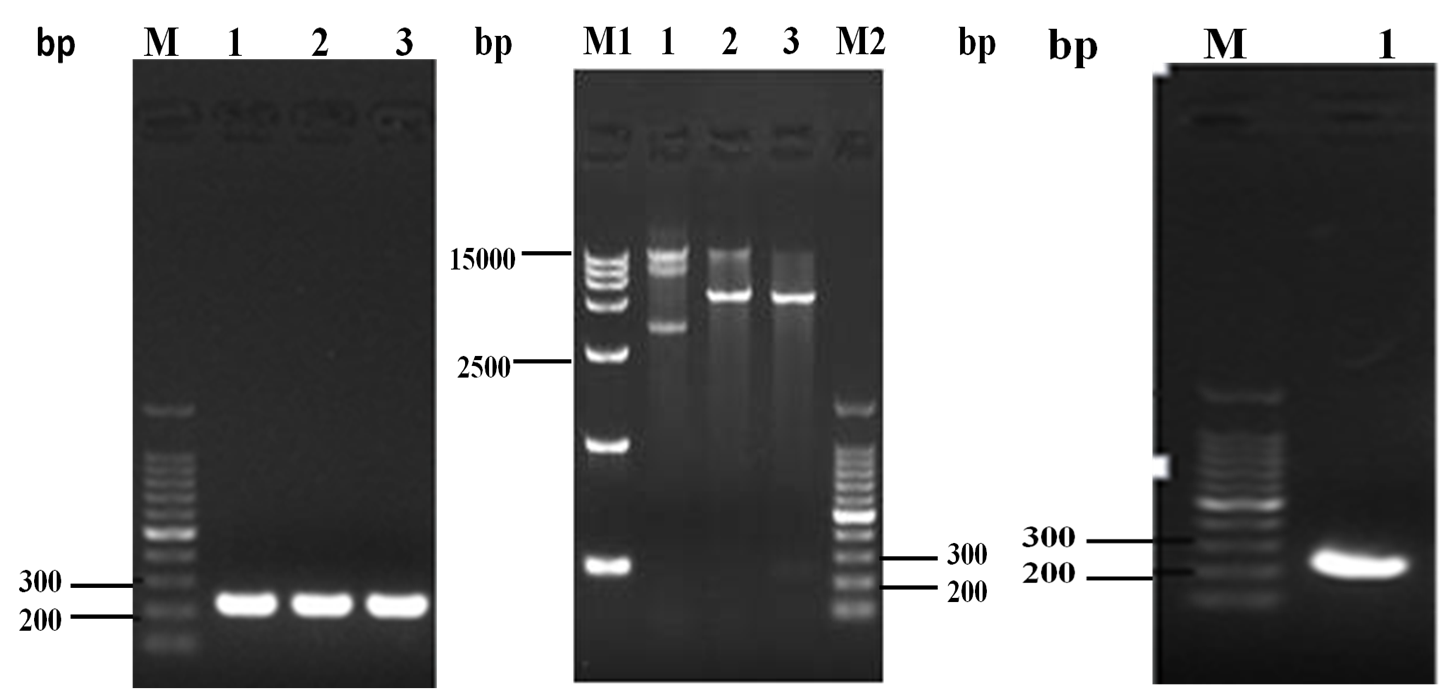** |
| --- |
| a b c  **Supplementary Figure 6. Cloning and identification of bacteriocin gene *Bt*CspB.** (a) PCR amplification of bacteriocin gene *Bt*CspB. M：100 bp plus DNA ladder purchased from TaKaRa BIO Inc. (Dalian, China)；1-3：*Bt*CspB PCR product; (b) Digestion analysis of recombinant plasmid pET32a-*Bt*CspB. M1：DL15000 marker purchased from TaKaRa BIO Inc. (Dalian, China)；M2：100 bp plus DNA ladder purchased from TaKaRa BIO Inc. (Dalian, China)；1: pET32a-*Bt*CspB; 2：*Xho*I digested pET32a-BtCspB; 3: *Nde*I and *Xho*I digested pET32a-*Bt*CspB; (c) PCR identification of recombinant plasmid pET32a-*Bt*CspB. M：100 bp plus DNA ladder purchased from TaKaRa BIO Inc. (Dalian, China)；1: *Bt*CspB PCR product. |

**Supplementary Table 1 Bt strains used in this study**

| Isolation  sources | Specific item for isolation | Sampling site*^a^* | Bt strain name |
| --- | --- | --- | --- |
| food | milk | Supermarket A, Fuzhou, Fujian | BRC-LJ2 |
|  | milk | Supermarket A, Fuzhou, Fujian | BRC-SFR29 |
|  | milk | Supermarket B, Fuzhou, Fujian | BRC-XQ9*^c^*, BRC-XQ10, BRC-XQ11*^b,c^*, BRC-XQ12 |
|  | bread | Supermarket A, Fuzhou, Fujian | BRC-SFR25, BRC-SFR26 |
|  | bread | Supermarket A, Fuzhou, Fujian | BRC-SFR27, BRC-SFR28 |
|  | jelly | Supermarket A, Fuzhou, Fujian | BRC-LJ6 |
|  | jelly | Supermarket A, Fuzhou, Fujian | BRC-SFR30 |
|  | jelly | Supermarket A, Fuzhou, Fujian | BRC-XQ1 |
|  | jelly | Supermarket A, Fuzhou, Fujian | BRC-XQ3, BRC-XQ4 |
|  | jelly | Supermarket A, Fuzhou, Fujian | BRC-XQ8*^c^* |
|  | jerry | Supermarket B, Fuzhou, Fujian | BRC-XQ14, BRC-XQ15 |
|  | jerry | Supermarket B, Fuzhou, Fujian | BRC-XQ16, BRC-XQ17 |
|  | jerry | Supermarket B, Fuzhou, Fujian | BRC-XQ18*^c^*, BRC-XQ19 |
| plant | *Michelia alba* | Supermarket B, Fuzhou, Fujian | BRC-LLP29 |
|  | *Michelia alba* | Wuyishan, Nanping, Fujian | BRC-ZLL7 |
|  | bryophyta | Wuyishan, Nanping, Fujian | BRC-SFR2, BRC-SFR4, BRC-SFR20, BRC-SFR21, BRC-SFR22, BRC-SFR23, BRC-SFR24*^c^* |
|  | bamboo leaf | Wuyishan, Nanping, Fujian | BRC-SFR5 |
|  | *Dicranopteris linearis* | Wuyishan, Nanping, Fujian | BRC-SFR6, BRC-SFR15 |
|  | leaf | Wuyishan, Nanping, Fujian | BRC-SFR7, BRC-ZXJ2*^c^* |
|  | *Bauhinia* | Fujian Agriculture and Forestry University, Fuzhou, Fujian | BRC-ZLL2 |
|  | *Caryota* | Fuzhou national forest park, Fuzhou, Fujian | BRC-ZLL5 |
|  | lichen | Gushan, Fuzhou, Fujian | BRC-ZQL1, BRC-ZQL2, BRC-ZQL3, BRC-ZQL4, BRC-ZQL5, BRC-ZQL6 |
| soil | soil | Sanming chemical factory, Fuzhou, Fujian | BRC-HZM3*^c^*, BRC-HZM5*^c^*, BRC-HZM7*^c^* |
|  | soil | Wuyishan, Nanping, Fujian | BRC-HZP2, BRC-HZP3, BRC-HZP5, BRC-HZP7, BRC-HZP9, BRC-HZP12*^c^*, BRC-GYH1*^b^*, *^c^*, BRC-ZXJ1 *^b, c^* |
|  | soil | Tianma Mountain, Mutian, Fujian | BRC-WSQ1*^b^* |
|  | uranium-  contaminated soil | Xinjiang | BRC-ZYR2*^b^*, *^c^*, BRC-ZYR3*^c^* |
| water | water | Fuzhou national forest park, Fuzhou, Fujian | BRC-CWS1 |
|  | water | Sanming chemical factory, Fuzhou, Fujian  Sanming pesticide factory, Fuzhou, Fujian | BRC-HZM1*^c^*, BRC-HZM2*^c^* |
|  | water | Putian city waste water, Fujian | BRC-WLY1 *^c^* |
|  | water | Wuyishan, Nanping, Fujian | BRC-SFR8, BRC-SFR9, BRC-SFR10*^c^*, BRC-SFR11, BRC-SFR12, BRC-SFR14*^b, c^* |
| feces | spotted deer | Fuzhou zoo, Fujian | BRC-WCB2 |
|  | spotted deer | Fuzhou zoo, Fujian | BRC-WCB9 |
|  | giant panda | Fuhzou panda world, Fujian | BRC-WCB3, BRC-WCB5, BRC-WCB6 |
|  | *Budorcas taxicolor* | Fuzhou zoo, Fujian | BRC-WCB10 |
|  | *Siberian tiger* | Sanming zoo, Fujian | BRC-WCB11 |
|  | horse | Sanming zoo, Fujian | BRC-WCB12 |
|  | peafowl | Sanming zoo, Fujian | BRC-WCB13 |
|  | macaque | Sanming zoo, Fujian | BRC-WCB14 |
| other | fodder | Xiayang fodder factory, Nanping, Fujian | BRC-ZYR1 |

*^a^*All the places are located at People’s Republic of China.

*^b^*Strains active against *B. cereus* 0938.

*^c^*Strains active against *B. cereus* ATCC 10987.

| **Supplementary Table 2** Indicator strains used in this study | | | |
| --- | --- | --- | --- |
| Characteristics | Species | Strain name^a^ | Incubation condition |
| Gram positive (G^+^) | *Listeria monocytogenes* | 0908, 0910, 0915, 0917, 100522, 100525, 100526, ATCC 15313, CMCC 54004 | TSB-30 ºC |
|  | *Listeria seeligeri* | 100521 | TSB-30 ºC |
|  | *Listeria inoccua* | 100523 | TSB-30 ºC |
|  | *Listeria ivanovii* | 100524 | TSB-30 ºC |
|  | *Bacillus cereus* | 0938, ATCC 10987 | TSB-30 ºC |
|  | *Enterococcus faecali* | a, c | TSB-30 ºC |
|  | *Enterococcus urinary* | b, d | TSB-30 ºC |
|  | *Enterococcus faecium* | ATCC 27270 | TSB-30 ºC |
|  | *Micrococcus luteus* | a | TSB-30 ºC |
|  | *Staphylococcus aureus* | ATCC 25923, ATCC 29213,  080202 | TSB-30 ºC |
|  | *Staphylococcus* *epidermidis* | CMCC(B) 26069 | TSB-30 ºC |
| Gram  negative  (G^-^) | *Escherichia coli* | ATCC 10536, ATCC 8739, ATCC 25922, O157:H7, K88, K12, 1739 | TSB-30 ºC |
|  | *Salmonella enterica* subsp. *enterica* serovar *Enteritidis* | ATCC 13076, a | TSB-30 ºC |
|  | *Salmonella enterica* subsp. *enterica* serovar *Typhimurium* | ATCC 13311 | TSB-30 ºC |
|  | *Salmonella paratyphi* C | a | TSB-30 ºC |
|  | *Salmonella* | W090914, 090022, 090023, 090024 | TSB-30 ºC |
|  | *Proteus mirabilis* | a | TSB-30 ºC |
|  | *Pseudomonas aeruginosa* | ATCC 15442  ATCC 9027 | TSB-30 ºC |
|  | *Yersinia enterocolitica* | a | TSB-30 ºC |
|  | *Shigella dysenteriae* | CMCC 51252 | TSB-30 ºC |
|  | [*Shigella flexneri*](http://www.google.ca/url?sa=t&rct=j&q=&esrc=s&frm=1&source=web&cd=1&cad=rja&ved=0CCUQFjAA&url=http%3A%2F%2Fdict.youdao.com%2Fw%2Fshigella_flexneri%2F&ei=DhjfUoWJLIS1qgGG3IG4DQ&usg=AFQjCNFxX1EoJowYgkRCsr600SbiFwYwcw&bvm=bv.59568121,d.aWc) | ATCC 12022 | TSB-30 ºC |
|  | *Shigella sonnei* | CMCC(B) 51592 | TSB-30 ºC |
|  | *Vibrio parahaemolyticus* | ATCC 17802, CMCC 17202 | TSB-30 ºC |
|  | *Vibrio vulnificus* | ATCC 27562 | TSB-30 ºC |

*^a^*They were identified to species by Fuzhou Center for Diseases Control and Prevention (China) according to the National Standards of the People's Republic of China.

| **Supplementary Table 3** Purification results of Bt BRC-ZYR2 bacteriocins | | | | |
| --- | --- | --- | --- | --- |
| Purification steps | Volume (mL) | Protein concentration（μg/ml） | Activity (U) | Recovery rate (%) |
| 100% saturation ammonium sulfate | 68 | 1364.2 | 608.2 | 100 |
| Sephadex G-50 column | 30 | 657.5 | 550.5 | 90.5 |
| 1st round of DEAE-52 cellulose column | 15 | 323.8 | 466.9 | 76.8 |
| 2nd round of DEAE-52 cellulose column | 2.5 | 35.6 | 235.7 | 38.8 |
